# Supplementary material for: A phenome-wide association study of genetically determined nicotine metabolism reveals novel links with health-related outcomes
Source: Eur J Epidemiol. 2025 Jul 12;40(9):1045–65. doi: 10.1007/s10654-025-01270-5 (PMC12537601; doi:10.1007/s10654-025-01270-5)
Supplement: Supplementary file 9 — Supplementary Information (pdf 12867 KB) [file 10654_2025_1270_MOESM9_ESM.pdf]

Supplementary Information

A Phenome-wide association study of genetically determined nicotine metabolism reveals novel links with health-related outcomes

Jadwiga Buchwald[1*], Terho Lehtimäki[2,3], Olli Raitakari[4,5,6], Veikko Salomaa[7], Jaakko Kaprio[1], Matti Pirinen[1,8,9]

1 Institute for Molecular Medicine Finland (FIMM), Helsinki Institute of Life Science (HiLIFE), University of Helsinki, Helsinki, Finland
2 Department of Clinical Chemistry, Fimlab Laboratories, and Finnish Cardiovascular Research Center, Tampere, Finland
3 Faculty of Medicine and Health Technology, Tampere, Finland
4 Centre for Population Health Research, University of Turku and Turku University Hospital, Turku, Finland
5 Research Centre of Applied and Preventive Cardiovascular Medicine, University of Turku, Turku, Finland
6 Department of Clinical Physiology and Nuclear Medicine, Turku University Hospital, Turku, Finland
7 Department of Public Health and Welfare, Finnish Institute for Health and Welfare, Helsinki, Finland
8 Department of Mathematics and Statistics, University of Helsinki, Helsinki, Finland
9 Department of Public Health, University of Helsinki, Helsinki, Finland

* jadwiga.buchwald@helsinki.fi

Contents

1 Supplementary Figures **3**

2 Supplementary Tables **13**

3 Extended Content **18**

3.1 FINEMAP analyses 18

3.2 The GxE MR-pheWAS approach 18

1 Supplementary Figures

List of Figures

1 **Fig S1.** Pipeline plot summarising the majority of methods and UKB data used. Main analyses are in pink and follow-up analyses in blue. *GS*, Genetic Score; *PWS*, Phenome-wide significant; *NMR*, Nicotine Metabolite Ratio; *CPD*, Cigarettes smoked per day; *10 PCs*, first ten principal components of genetic structure. 4

2 **Fig S2.** Boxplots of the standardised GS for the NMR in UKB by subgroups. Experimenters includes those individuals who answered "Occasionally" or "Tried once or twice" to the questions on current and past smoking behaviour. 5

3 **Fig S3.** Scatterplot of the GS for faster nicotine metabolism against the imputed genotype dosage (allele C) at the chromosome 19 top SNP (rs56113850) for the NMR in UKB. . . 5

4 **Fig S4.** Scatterplots and loess curves presenting the full data of the association between **(A)** the standardized genetic score for the NMR (zGS) and cigarettes smoked per day (CPD) in UKB, **(B)** the standardized NMR (zNMR) and CPD in the Finnish data, **(C)** the NMR and CPD in the Finnish data, and **(D)** the zNMR and Cotinine + 3-Hydroxycotinine (Cot + 3HC), a biomarker for nicotine intake, in the Finnish data. All plots are for current smokers. The standardized variables (zGS and zNMR) were calculated by subtracting the mean and dividing by the standard deviation. 6

5 **Fig S5.** Venn diagram of the 61 variables highlighted in our initial PheWAS. The figure shows the 61 variables that were statistically significant at the 5 % FDR level in at least one of the data sets (All / Ever / Never). The variable Smoking status has been listed twice in the figure as it contained a different amount of categories for the All and Ever groups. 7

6 **Fig S6.** Forest plot of the liver enzyme associations, showing the original results and those obtained after additionally adjusting for alcohol intake frequency. All results were phenome-wide significant (PWS), i.e. in All p-values $< 1.1e-04$, in Ever p-values $< 8.7e-05$, and in Never p-values $< 6.2e-06$. 8

7 **Fig S7.** Forest plot of the 18 variables highlighted in our sex-stratified analyses of the 71 variables that were included in our final PheWAS. Results for males have been indicated with squares and results for females with diamonds. Solid circles/squares indicate a statistically significant effect size at $p < 0.05$. The figure shows the variables that had a statistically significant ($p < 0.05$) difference between the effect sizes of females and males in at least one of the data groups (Never/Ever/All). *, there was a Bonferroni significant ($p < 0.05/71$) difference between the effect sizes of the males and females; *n*, normalised after covariates had first been regressed out; *d*, derived from the original UKB phenotype; *c*, coding corrected to be more intuitive. 9

8 **Fig S7** continued 10

9 **Fig S7** continued 11

10 **Fig S8.** Forest plot of the 11 variables highlighted in our ancestry-stratified analysis of the 33 continuous variables that were phenome-wide significant in our final PheWAS. The figure shows the 11 variables that were statistically significant ($p < 0.05$) in at least one of the ancestry groups (solid circles/squares), other than White British, or had a statistically significant difference in their effect sizes as compared to the White British group ($p < 0.05$) (square shape). *n*, normalised after covariates had first been regressed out; *d*, derived from the original UKB phenotype. 12

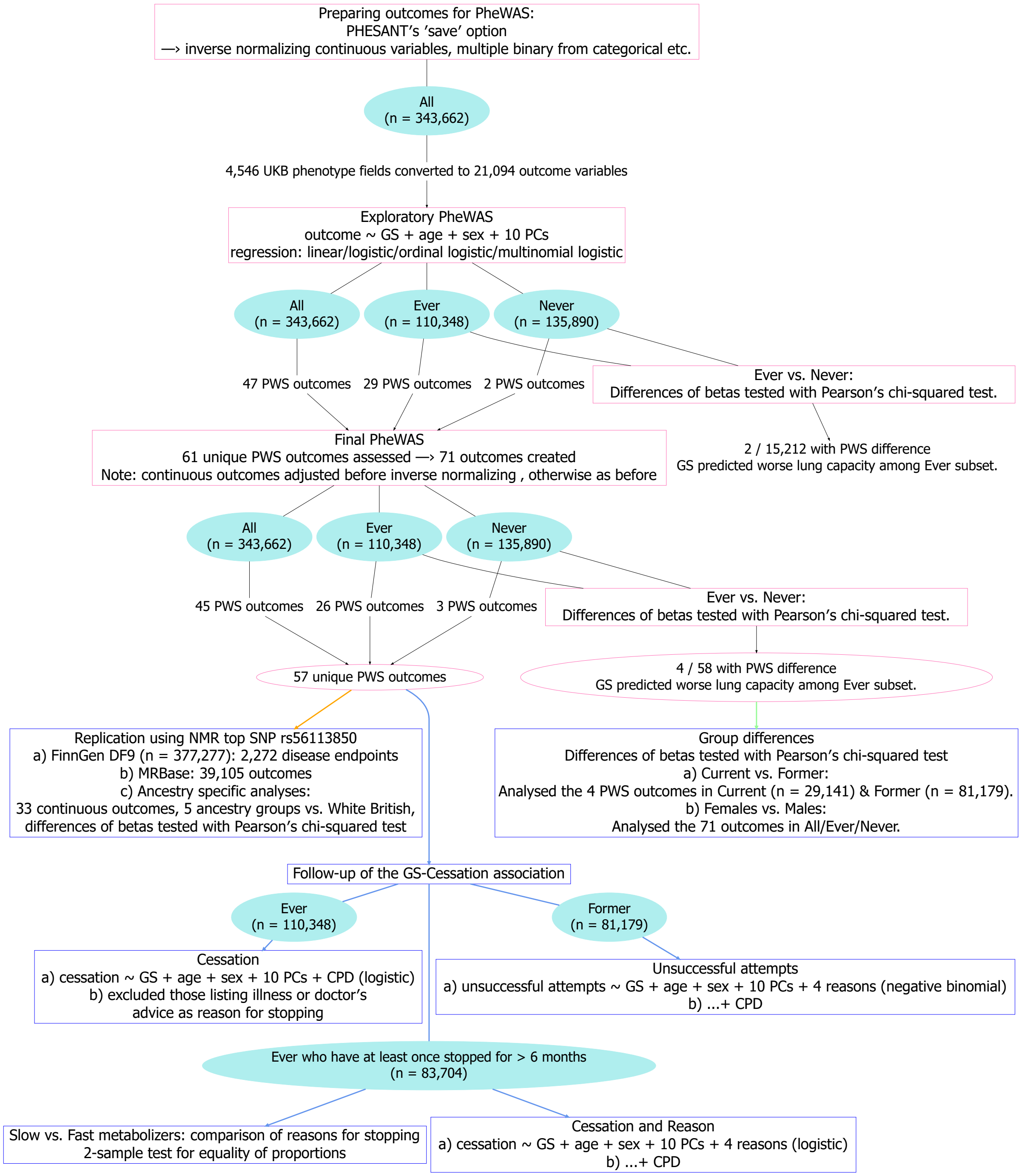

Fig S1. Pipeline plot summarising the majority of methods and UKB data used. Main analyses are in pink and follow-up analyses in blue. *GS*, Genetic Score; *PWS*, Phenome-wide significant; *NMR*, Nicotine Metabolite Ratio; *CPD*, Cigarettes smoked per day; *10 PCs*, first ten principal components of genetic structure.

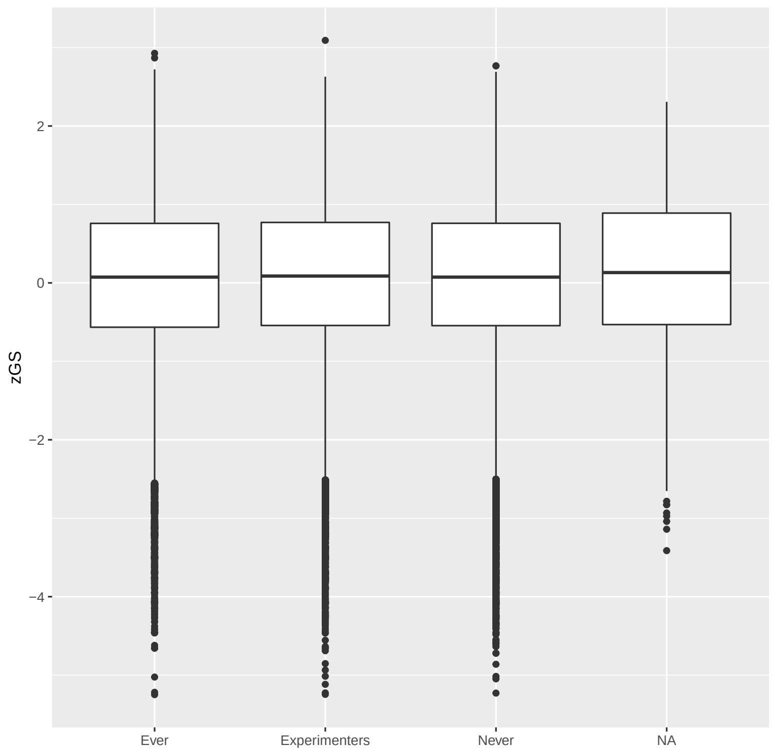

Fig S2. Boxplots of the standardised GS for the NMR in UKB by subgroups. Experimenters includes those individuals who answered "Occasionally" or "Tried once or twice" to the questions on current and past smoking behaviour.

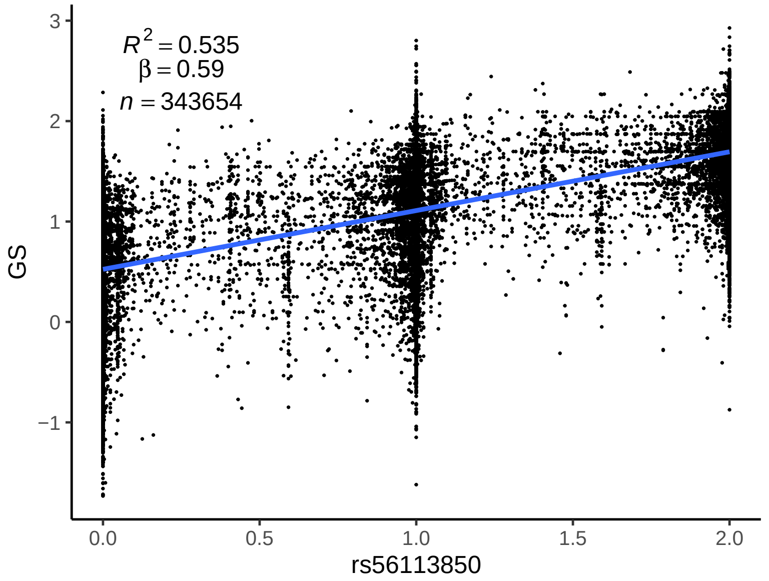

Fig S3. Scatterplot of the GS for faster nicotine metabolism against the imputed genotype dosage (allele C) at the chromosome 19 top SNP (rs56113850) for the NMR in UKB.

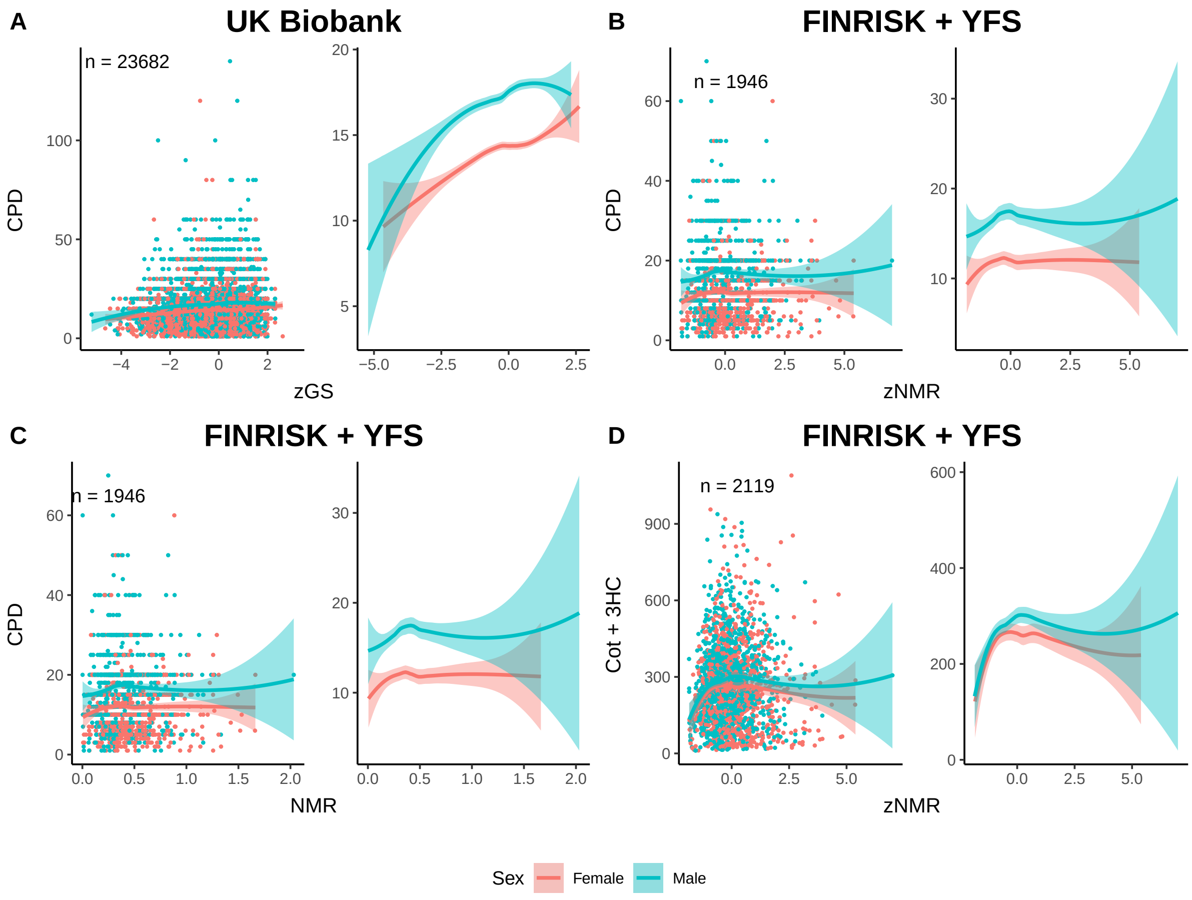

Fig S4. Scatterplots and loess curves presenting the full data of the association between **(A)** the standardized genetic score for the NMR (zGS) and cigarettes smoked per day (CPD) in UKB, **(B)** the standardized NMR (zNMR) and CPD in the Finnish data, **(C)** the NMR and CPD in the Finnish data, and **(D)** the zNMR and Cotinine + 3-Hydroxycotinine (Cot + 3HC), a biomarker for nicotine intake, in the Finnish data. All plots are for current smokers. The standardized variables (zGS and zNMR) were calculated by subtracting the mean and dividing by the standard deviation.

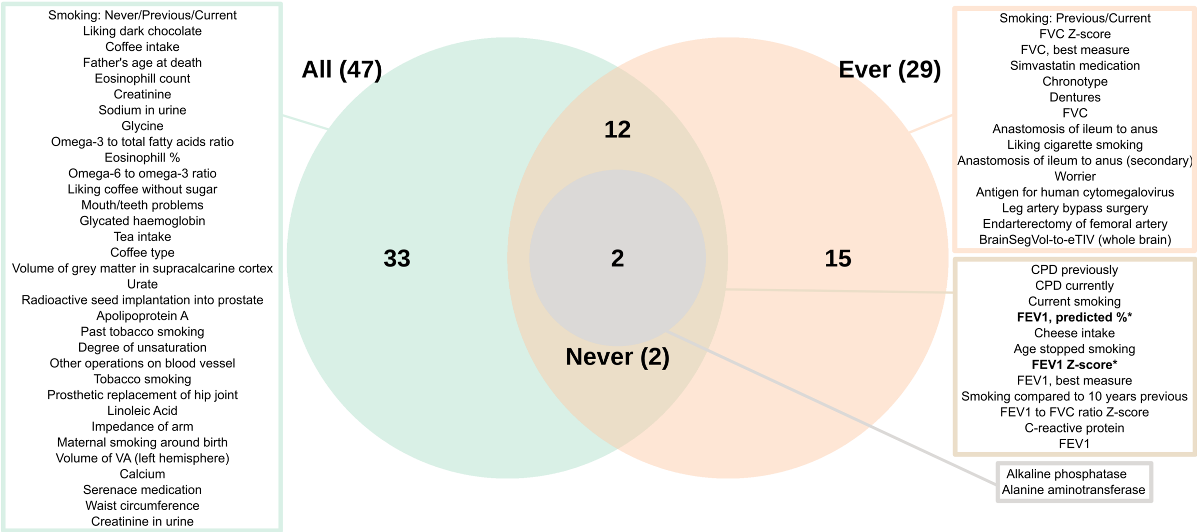

Fig S5. Venn diagram of the 61 variables highlighted in our initial PheWAS. The figure shows the 61 variables that were statistically significant at the 5 % FDR level in at least one of the data sets (All / Ever / Never). The variable Smoking status has been listed twice in the figure as it contained a different amount of categories for the All and Ever groups.

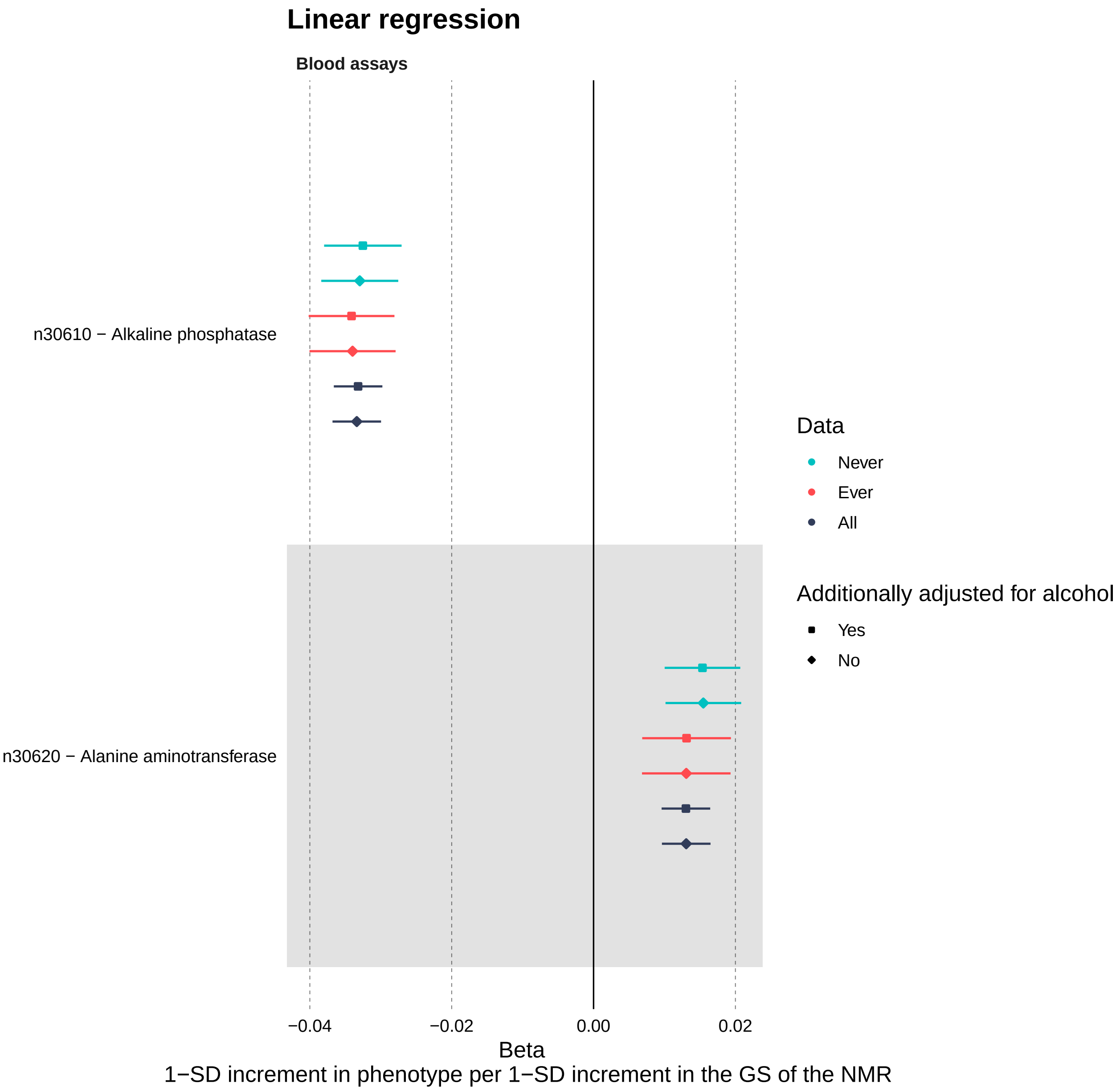

Fig S6. Forest plot of the liver enzyme associations, showing the original results and those obtained after additionally adjusting for alcohol intake frequency. All results were phenome-wide significant (PWS), i.e. in All p-values $< 1.1e-04$, in Ever p-values $< 8.7e-05$, and in Never p-values $< 6.2e-06$.

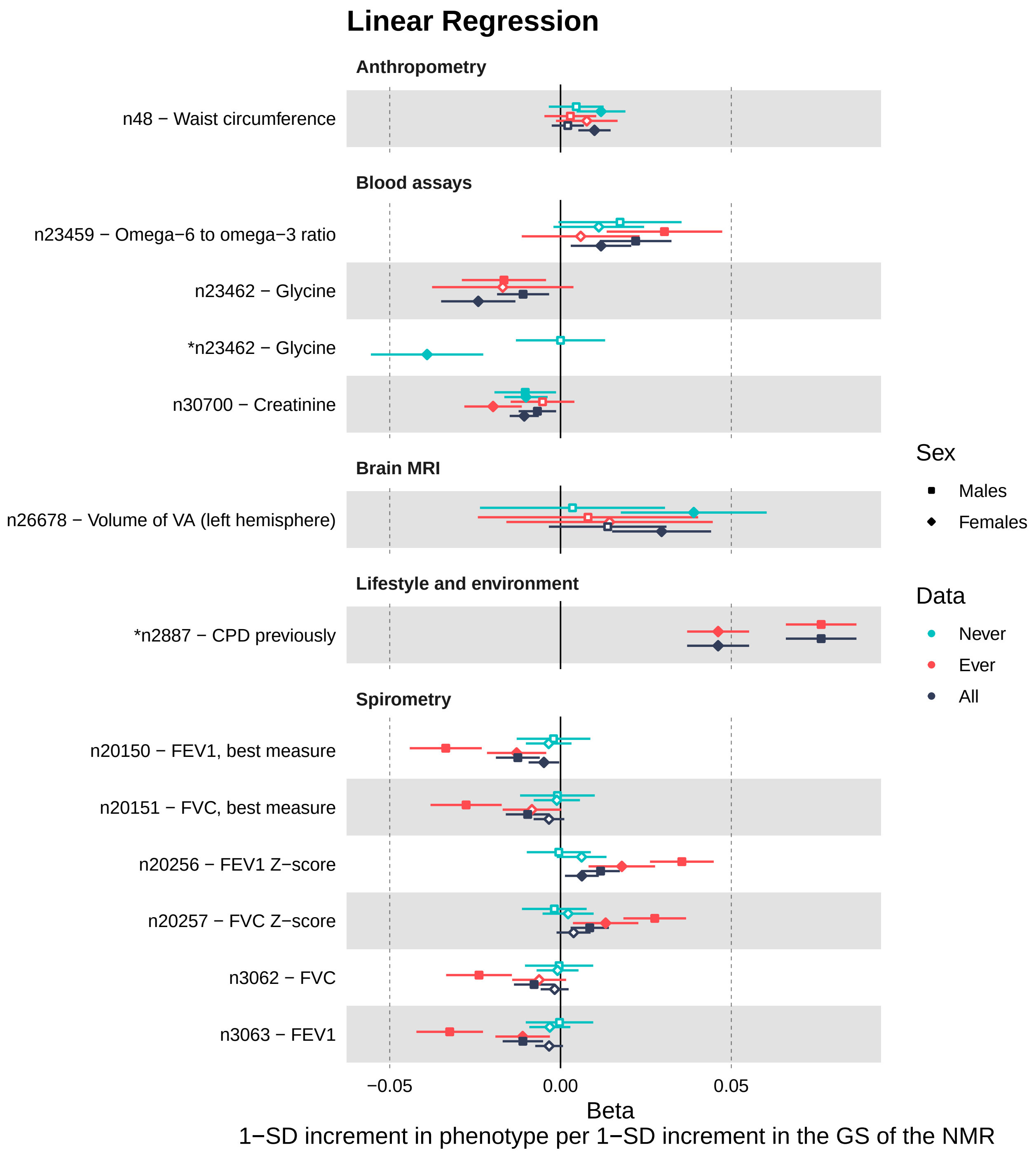

Fig S7. Forest plot of the 18 variables highlighted in our sex-stratified analyses of the 71 variables that were included in our final PheWAS. Results for males have been indicated with squares and results for females with diamonds. Solid circles/squares indicate a statistically significant effect size at $p < 0.05$. The figure shows the variables that had a statistically significant ($p < 0.05$) difference between the effect sizes of females and males in at least one of the data groups (Never/Ever/All). *, there was a Bonferroni significant ($p < 0.05/71$) difference between the effect sizes of the males and females; n, normalised after covariates had first been regressed out; d, derived from the original UKB phenotype; c, coding corrected to be more intuitive.

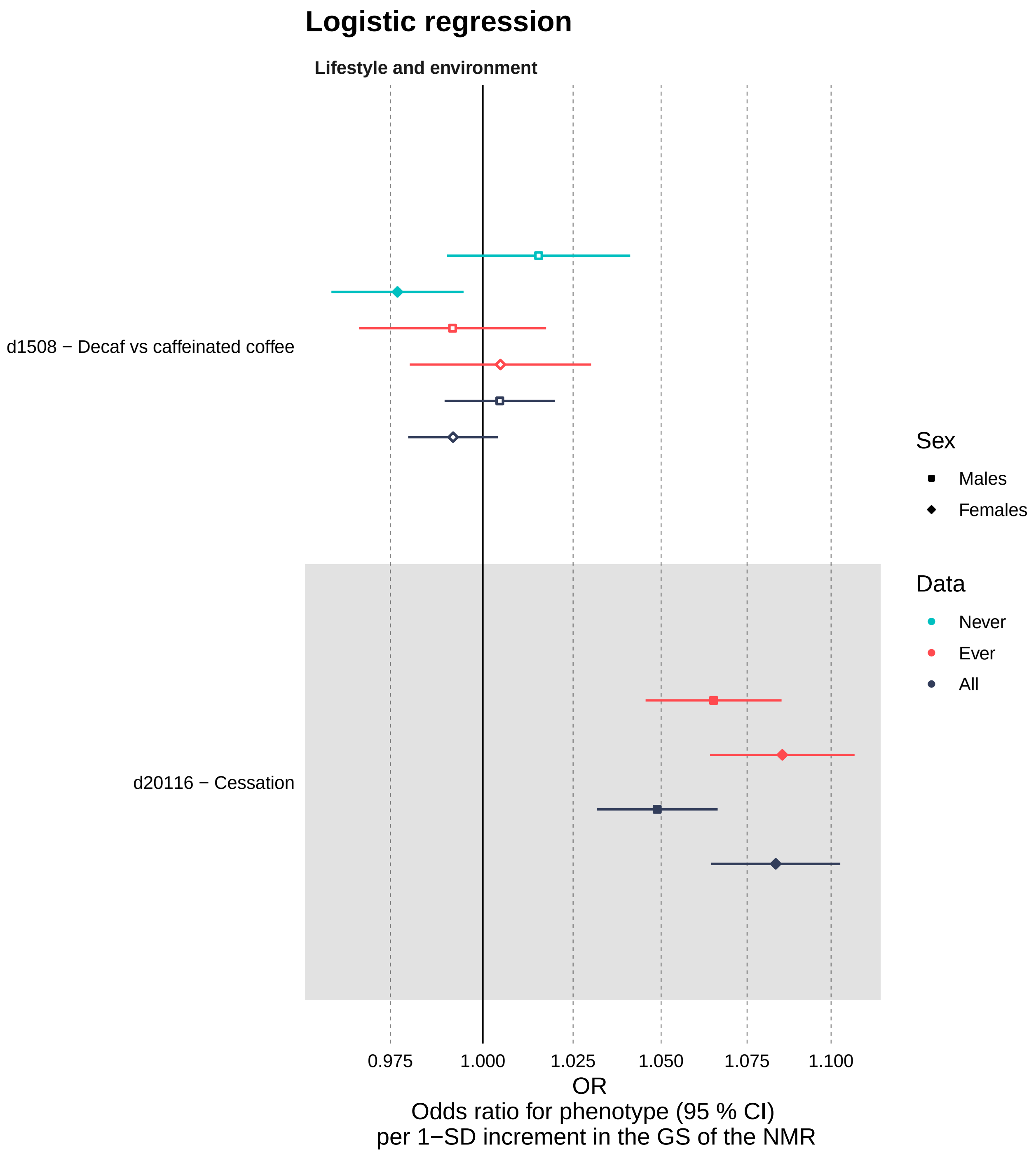

Fig S7 continued

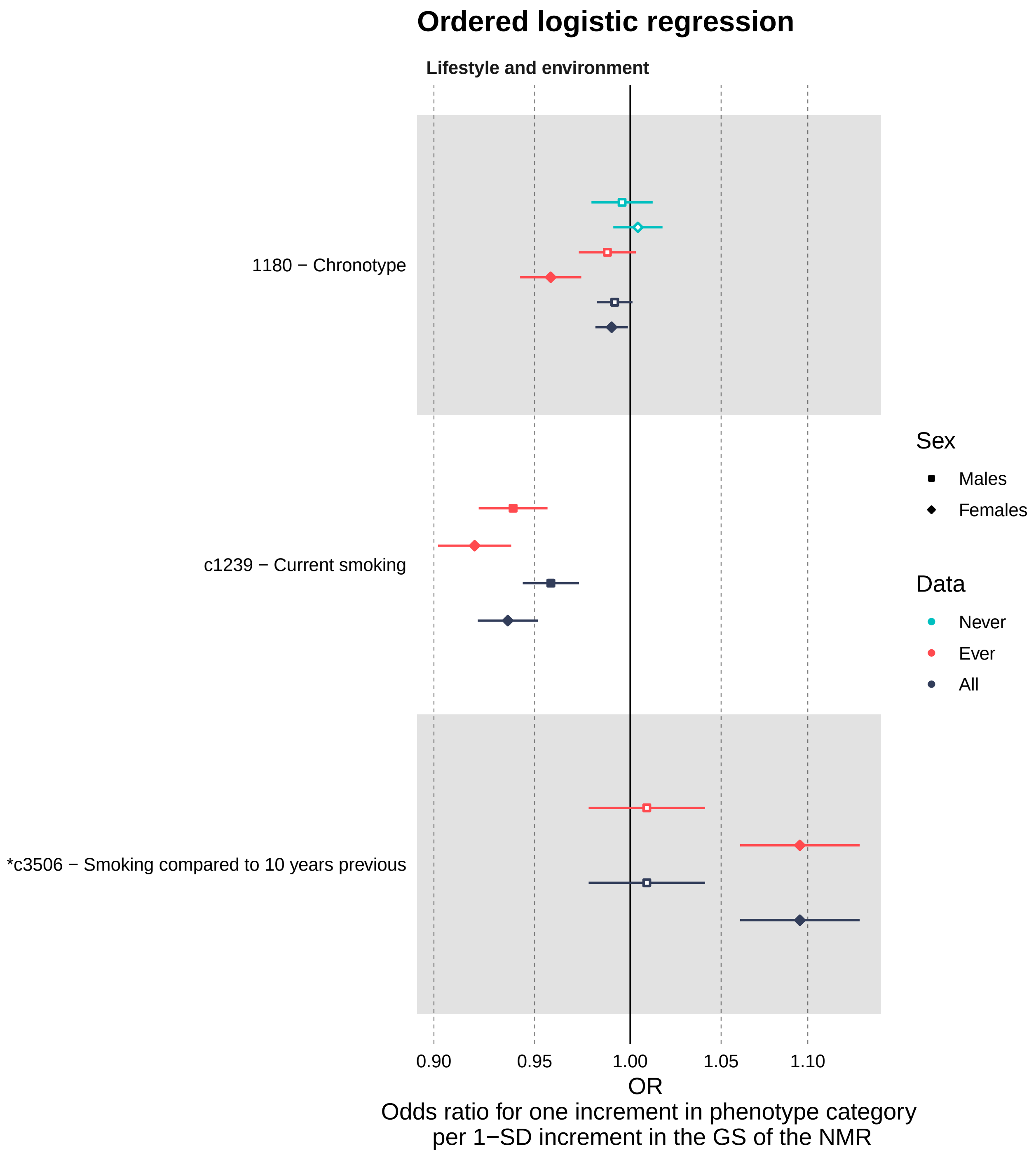

Fig S7 continued

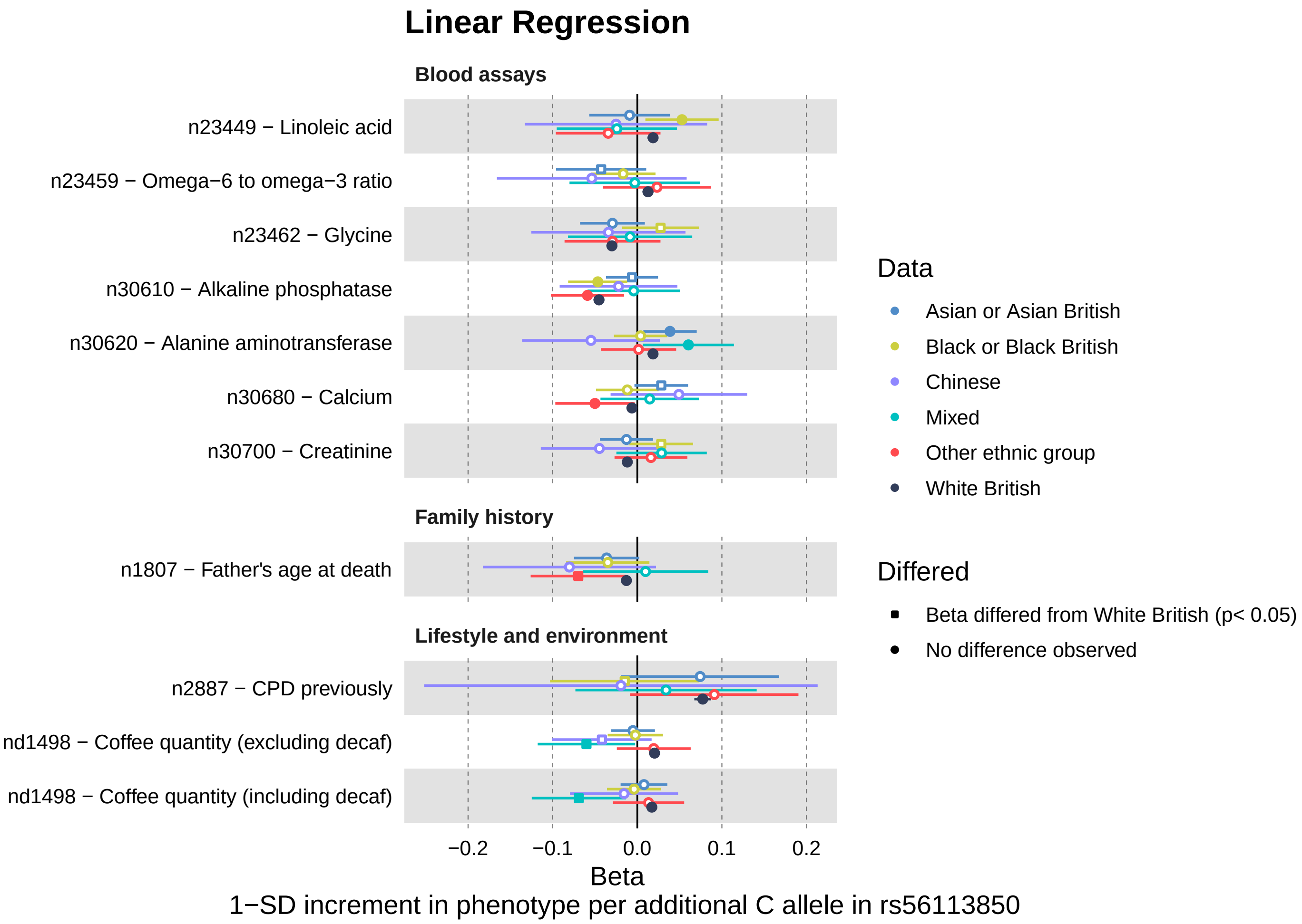

Fig S8. Forest plot of the 11 variables highlighted in our ancestry-stratified analysis of the 33 continuous variables that were phenome-wide significant in our final PheWAS. The figure shows the 11 variables that were statistically significant ($p < 0.05$) in at least one of the ancestry groups (solid circles/squares), other than White British, or had a statistically significant difference in their effect sizes as compared to the White British group ($p < 0.05$) (square shape). n, normalised after covariates had first been regressed out; d, derived from the original UKB phenotype.

2 Supplementary Tables

List of Tables

1 **Table S1.** GS distribution by subsets of smoking status 14
2 **Table S2.** FINEMAP top configuration of causal SNPs for the NMR on the chromosome 19 locus in the Finnish data 15
3 **Table S4.** Linear regression beta coefficient for the zGS when explaining CPD by zGS in UKB 15
4 **Table S8.** Reason for stopping smoking by nicotine metabolism group (grouped based on the tertiles of the GS for the NMR) 16
5 **Table S10.** Results of the liver enzyme associations, showing the original results and those obtained after additionally adjusting for alcohol intake frequency 16
6 **Table S12.** Summary of rs56113850 association results across NMR GWAS studies performed to date 17
7 **Table S13.** Information on the NMR top SNP, rs56113850, and smoking status by ancestry group 17

List of Excel Tables Provided Separately

Supplementary Tables 3, 5, 6, 7, 9, 11, 14 & 15 are provided as excel tables seperately.

Table S3 Variable descriptions for PheWAS outcomes. A Variable information on all the second stage outcomes that we derived, recoded or analysed using a different model from our initial PheWAS (See Table S3b). **B** Variable information on all 61 variables highlighted in our initial PheWAS analyses.

Table S5 Results from the initial PheWAS. A-C PheWAS results for the variables that were significant at the 5 % FDR level for the All, Ever and Never data, respectively. **D** Ever versus Never analysis results for the variables that had a statistically significant difference at the 5 % FDR level between their effect sizes for the Ever and Never subsets.

Table S6 Results from the final stage of the PheWAS. A Ever versus Never analysis results. **B-D** PheWAS results for the All, Ever and Never data, respectively. **E** Current versus Former analysis results for the four lung capacity measures that had been highlighted in the Ever versus Never analysis (See Table S6a).

Table S7 Odds ratios from the logistic regression model for cessation. A Among Ever smokers (n = 110348), model including the standardized GS, sex (1=Male, 0=Female), age and the first 10 genetic principal components as the predictor variables. **B** Among Ever smokers (n = 110348), model including CPD as an additional predictor variable. **C** Among subset of Ever smokers who did not stop smoking due to Illness or Doctor's advice (n = 70278), model including all the same variables as in B. **D** Among subset of Ever smokers who had at least once managed to quit for over 6 months (n = 83704), model including the 4 reasons for stopping smoking. **E** Among subset of Ever smokers who had at least once managed to quit for over 6 months (n = 79228), model including the 4 reasons for stopping smoking and CPD.

Table S9 Incident Rate Ratios (IRRs) from the negative binomial regression model for Number of unsuccessful stop-smoking attempts. A Among former smokers, model including standardized GS, the four reasons (1 = Yes, 0 = No), sex (1 = Male, 0 = Female), age and the first 10

genetic principal components as the predictor variables. **B** Same as A but including CPD as a covariate in the model.

Table S11 The FDR significant results from the PheWAS of the top NMR SNP, rs56113850 (allele C), using FinnGen and MRBase. **A** Using the FinnGen r9 there were two outcomes reaching statistical significance at the 0.05 FDR level. Beta has been counted for the C allele. Allele C frequency (vs T) was 56–57 % across all phenotypes. *mlogp*,-log10(p); *Bhcritical*, Benjamini-Hochberg critical value. **B** Using the MRBase (Database version: 0.3.0 from 25 October 2020) there were 199 outcomes reaching statistical significance at the 0.05 FDR level. *minuslogp*,-log10(p); *Bhcritical*, Benjamini-Hochberg critical value; *bf*, significant at the 0.05 bonferroni level (T=TRUE, F=FALSE). **C** Same as B but ordered by trait. **D** Annotations for the 14 gene expression outcomes that were among the 199 MRBase results. Annotations were obtained from the Ensembl database using BiomaRt in R. *band*, Karyotype band; *gene_biotype*, Gene type; *hgnc_symbol*, HGNC (The HUGO Gene Nomenclature Committee) gene symbol. Note: MRBase id column can be searched as the GWAS ID here: https://gwas.mrcieu.ac.uk/datasets/ for more information on the study.

Table S14 Sex-stratified analyses. Results for all 71 variables assessed.

Table S15 Ancestry-stratified analyses. Results for all 33 continuous variables assessed.

Table S1. GS distribution by subsets of smoking status

	n	minimum	maximum	median	mean	sd	p-value
Never	135890	-1.721	2.746	1.241	1.198	0.559	-
Ever	110348	-1.733	2.835	1.241	1.196	0.561	0.4511
"Occasionally" or "Once or twice"	96272	-1.731	2.927	1.249	1.207	0.555	0.0009
NA	1152	-0.707	2.489	1.273	1.223	0.557	0.0945

n, number of observations; *sd*, standard deviation; *NA*, missing. The p-values are from Mann-Whitney U tests comparing the GS distributions to the Never group.

Table S2. FINEMAP top configuration of causal SNPs for the NMR on the chromosome 19 locus in the Finnish data

SNP	BP	EA/NEA	MINOR	MAF	SNP PROB	BETAJ	SEJ	P-VALUEJ
RS12985907	41343544	A/G	A	0.28	0.9998	-0.8	0.03	1.21×10^{-118}
RS189621498	41288136	A/G	A	0.03	0.9763	0.61	0.08	9.9×10^{-16}
RS1801272	41354533	T/A	T	0.02	0.9749	-1.01	0.08	6.13×10^{-34}
RS34945948	41340842	G/A	G	0.14	0.519	1.03	0.07	1×10^{-54}
RS7248187	41437426	C/G	G	0.24	0.4319	0.32	0.03	4.63×10^{-22}
RS116382863	41534881	T/C	T	0.13	0.1694	0.22	0.04	3.2×10^{-9}
RS11466310	41861858	T/C	T	0.02	0.0714	-0.54	0.1	4.18×10^{-8}
RS7250713	41355195	C/G	G	0.37	0.028	0.33	0.03	1.99×10^{-21}
RS74719953	41335799	T/C	T	0.09	0.0027	-0.36	0.07	8.23×10^{-8}

FINEMAP results of the 5Mb region centered at the top SNP when only including SNPs passing quality control in UKB (n(SNP) = 10,133). The most probable configuration consisted of 9 SNPs (depicted in the table) and their heritability estimate was 32.1% (95% CI: 28.5–35.6%). FINEMAP gave a regional heritability estimate of 33.8% (95% CI: 30.0–37.8%) and suggested that there are 7–11 causal SNPs within the region.
SNP, single-nucleotide polymorphism; *BP*, base pair position in GRCh37 coordinates; *EA/NEA*, the effect allele/ the non-effect allele; *MINOR*, the less common allele; *MAF*, minor allele frequency in the Finnish dataset (n = 2,119) used for the FINEMAP analysis; *SNP PROB*, posterior probability of being a causal SNP; *BETAJ*, effect estimate from the joint model including all these 9 SNPs (reported for the effect allele); *SEJ*, standard error for the BETAJ; *P-VALUEJ*, p-value for the BETAJ.

Table S4. Linear regression beta coefficient for the zGS when explaining CPD by zGS in UKB

Data	Number of observations	Beta coefficient	p-value
ALL	23,682	0.07516	$< 2 \times 10^{-16}$
LOW GS ($zGS < 0$)	10,871	0.10982	$< 2 \times 10^{-16}$
HIGH GS ($zGS \geq 0$)	12,811	0.04237	0.0235

Analysis was done for the current smokers subset of UKB. CPD has been adjusted for sex, age and the first 10 genetic principal components, and then inverse normalized (rank-based-inverse-normal-transformation) before regressing it on zGS. The standard deviation of the adjusted CPD was 8.3. When looking at all individuals, each standard deviation increase in the GS is associated with a 0.07516 standard deviation increase in CPD, i.e. a 0.6 increase in cigarettes smoked per day. The regression was ran for three subsets of the data including either all individuals (ALL), only those with lower zGS values (LOW GS; $zGS < 0$) or only those with higher zGS values (HIGH GS; $zGS \geq 0$).

Table S8. Reason for stopping smoking by nicotine metabolism group (grouped based on the tertiles of the GS for the NMR)

	Slow (n = 36,783)	Medium (n = 36,782)	Fast (n = 36,783)	p-value (slow vs fast)
Illness or ill health	9.02 %	8.76 %	9.24 %	0.3243
Doctor's advice	5.38 %	5.35 %	5.50 %	0.5051
Health precaution	47.22 %	47.86 %	48.00 %	0.0360*
Financial reasons	18.36 %	18.97 %	19.24 %	0.0024*

Ever smokers who had stopped smoking for over 6 months during the time they smoked were asked: "Why did you stop smoking? (You can select more than one answer)" (UK Field 6157). Total number of ever smokers included in this analysis was $n = 110,348$, of which 26 % were current smokers and 74 % former smokers. *, Statistically significant difference ($p < 0.05$, 2-sample test for equality of proportions) between the Slow and Fast groups.

Table S10. Results of the liver enzyme associations, showing the original results and those obtained after additionally adjusting for alcohol intake frequency

Phenotype	Beta	Standard error	p-value	Analysis Type	Data Group
n30610 - Alkaline phosphatase	-0.0334	0.0017	2.1e-81	Basic	All
n30610 - Alkaline phosphatase	-0.0332	0.0017	1.6e-80	Adjusted	All
n30610 - Alkaline phosphatase	-0.034	0.0031	5.4e-28	Basic	Ever
n30610 - Alkaline phosphatase	-0.0341	0.0031	2.1e-28	Adjusted	Ever
n30610 - Alkaline phosphatase	-0.033	0.0028	1.2e-32	Basic	Never
n30610 - Alkaline phosphatase	-0.0325	0.0028	1.8e-31	Adjusted	Never
n30620 - Alanine aminotransferase	0.0131	0.0017	7.8e-14	Basic	All
n30620 - Alanine aminotransferase	0.013	0.0017	9.4e-14	Adjusted	All
n30620 - Alanine aminotransferase	0.0131	0.0032	4.1e-05	Basic	Ever
n30620 - Alanine aminotransferase	0.0131	0.0032	4e-05	Adjusted	Ever
n30620 - Alanine aminotransferase	0.0155	0.0027	1.3e-08	Basic	Never
n30620 - Alanine aminotransferase	0.0154	0.0027	1.6e-08	Adjusted	Never

All results were phenome-wide significant (PWS), i.e. in All p-values $< 1.1e-04$, in Ever p-values $< 8.7e-05$, and in Never p-values $< 6.2e-06$. *Basic*, analyses adjusted for age, sex, and the ten first principal components of genetic structure; *Adjusted*, analyses additionally adjusted for alcohol intake frequency.

Table S12. Summary of rs56113850 association results across NMR GWAS studies performed to date

Study	Population	Major/Minor	MAF	β_C	p-value	n
I (2015) [1]	FI META	C > T	0.45-0.46	0.65	5.77E-86	1518
Patel (2016) [2]	MIX META.	NA	NA	0.36	1.19E-50	2239
Baurley (2016) [3]	Eur.	C > T	0.32	0.33	3.81E-10	212
	Afr. Am.	C > T	0.49	0.40	5.22E-04	49
	As. Am.	T > C	0.42	0.36	1.62E-04	51
	MIX META	see above	0.32-0.49	0.34	6.61E-18	312
Chenoweth (2018) [4]	Afr. Am. META	NA	NA	0.45	5.31E-22	855
II (2021) [5]	Eur. META	C > T	0.45	0.68	5.54E-261	5185
Chenoweth (2021) [6]	Eur. F	C > T	0.48	0.67	7.5E-22	389
	Eur. M	C > T	0.45	0.75	1.2E-37	541
	Afr. Am. F	NA	NA	0.49	1.46E-15	503
	Afr. Am. M	NA	NA	0.43	1.28E-08	352

MAF, minor allele frequency; β_C, effect size for allele C; n, sample size; FI, Finnish; Eur. Am., European American; Afr. Am., African American; As. Am., Asian American; Eur., European Ancestry.

Table S13. Information on the NMR top SNP, rs56113850, and smoking status by ancestry group

Data	rs56113850: C (vs T) frequency	HW exact p-value	info	missing proportion	n	Smoking Status (%) Never/Previous/Current/NA
White British	0.57763	0.009	0.995	5.528e-10	343695	54.4 / 35.1 / 10.1 / 0.3
Mixed	0.511039	0.241	0.980	1.430e-08	2797	48.2 / 32.5 / 18.9 / 0.4
Asian	0.423935	8.525e-10	0.973	7.467e-09	9375	76.4 / 13.1 / 9.4 / 1.1
Black	0.388393	4.579e-05	0.972	1.313e-09	7618	69.7 / 17.2 / 12.3 / 0.8
Chinese	0.397583	0.001	0.900	5.988e-08	1503	78.8 / 13.3 / 7.6 / 0.3
Other ethnic group	0.483887	2.682e-10	0.965	6.890e-09	4354	60.7 / 25.3 / 13.3 / 0.7

n, number of observations; NA, missing.

3 Extended Content

3.1 FINEMAP analyses

The FINEMAP software [7] conducts a probabilistic variable selection analysis of GWAS results on a given genomic region. It is applied to the regions where genome-wide significant variants are found. The aim is to find the set of SNPs (so called "causal SNPs") that jointly best explain the association statistics observed across the whole region. As output, FINEMAP (version 1.4) provides 1) lists of plausible sets of causal SNPs with their probabilities, 2) for each SNP in the region, FINEMAP provides a posterior inclusion probability (PIP) that is the probability that the SNP is one of the causal SNPs in the region, and 3) for each independent association signal in the region, the 95% credible set lists the SNPs that are the most plausible candidates to be the causal SNP behind the association signal. As input, FINEMAP takes the GWAS results (effect sizes estimates and standard errors) and a pairwise SNP-by-SNP correlation matrix.

Already in our previous study [5] we performed FINEMAP analyses of the association loci (chromosome 4 and chromosome 19). In our previous analyses we used the GWAS results and the correlation matrix computed from the Finnish data, namely the meta-analysis of FINRISK and YFS (n = 2,119). Thus, we used the exact in-sample correlation matrix in the FINEMAP analysis (instead of using some reference data). The correlation matrix was computed using the LDstore software. Insertions and deletions were not included in our data as SNP data was required from both FINRISK and YFS, and the YFS data did not include insertions and deletions.

For this study, we reran the fine-mapping analyses of the chromosome 19 locus using a subset of the GWAS summary statistics and SNP correlation data previously used, which included SNPs within the ±2.5Mb flanking region of the top associating SNP (rs56113850). We included the SNPs passing the following quality control criteria in both the Finnish and the UKB data: imputation info score > 0.7, call rate > 0.9, and Hardy-Weinberg Equilibrium $p > 1 \times 10^{-6}$. Additionally, only the SNPs with minor allele frequency (MAF) > 1 % in the Finnish data were included and the multiallelic SNPs were excluded. Altogether, 10,133 SNPs were included in the FINEMAP analysis as opposed to the 12,060 SNPs included previously. We set the maximum number of allowed causal SNPs to 20, and otherwise used the default settings in FINEMAP (version 1.4). For the GS construction, we used the set of 9 SNPs that FINEMAP listed as being the most probable set of causal SNPs in the chromosome 19 locus.

3.2 The GxE MR-pheWAS approach

The method has been described in detail by Millard et al. [8]. In short, mendelian randomization (MR) uses a genetic instrument as a proxy for an exposure in order to test for a causal effect of the exposure on the outcome, while avoiding bias due to confounding or reverse causality. The key assumption is that the genetic instrument has an association with the outcome solely through the exposure. Most approaches available for testing whether there is evidence for horizontal pleiotropy, and thus, violation of this assumption, are only applicable when several independent genetic instruments proxying the exposure have been used. However, when multiple genetic instruments are not available, such as in our case where our genetic instrument is reliant on only two association loci, the gene-by-environment (GxE) design, can be used.

The idea in GxE MR is to divide the data in to groups with different levels of the exposure, in our case, ever and never smokers. We assume that the effect of nicotine metabolism only occurs in people who are actually using nicotine, in other words our ever smokers subset. Then, if a given association between our genetic instrument and outcome is only due to nicotine metabolism, either directly or indirectly through traits such as amount smoked, we should only see an association among ever smokers, but not among never smokers. The approach permits us to distinguish whether the associations reflect a causal pathway through a) the NMR, either directly or through other traits such as amount smoked (effect only seen in ever smokers), b) some other pathways not including the NMR (same effect seen also in never smokers), or c) both (effect only seen in never smokers, or there are quantitative or directional differences in the effect sizes between ever and never smokers) (see Figure 1).

References

1. Loukola A, Buchwald J, Gupta R, Palviainen T, Hällfors J, Tikkanen E, et al. A genome-wide association study of a biomarker of nicotine metabolism. PLoS genetics. 2015;11(9):e1005498.

2. Patel YM, Park SL, Han Y, Wilkens LR, Bickeböller H, Rosenberger A, et al. Novel association of genetic markers affecting CYP2A6 activity and lung cancer risk. Cancer research. 2016;76(19):5768–5776.

3. Baurley JW, Edlund CK, Pardamean CI, Conti DV, Krasnow R, Javitz HS, et al. Genome-wide association of the laboratory-based nicotine metabolite ratio in three ancestries. Nicotine & tobacco research. 2016;18(9):1837–1844.

4. Chenoweth MJ, Ware JJ, Zhu AZ, Cole CB, Cox LS, Nollen N, et al. Genome-wide association study of a nicotine metabolism biomarker in African American smokers: impact of chromosome 19 genetic influences. Addiction. 2018;113(3):509–523.

5. Buchwald J, Chenoweth MJ, Palviainen T, Zhu G, Benner C, Gordon S, et al. Genome-wide association meta-analysis of nicotine metabolism and cigarette consumption measures in smokers of European descent. Molecular psychiatry. 2021;26(6):2212–2223.

6. Chenoweth MJ, Cox LS, Nollen NL, Ahluwalia JS, Benowitz NL, Lerman C, et al. Analyses of nicotine metabolism biomarker genetics stratified by sex in African and European Americans. Scientific Reports. 2021;11(1):19572.

7. Benner C, Spencer CC, Havulinna AS, Salomaa V, Ripatti S, Pirinen M. FINEMAP: efficient variable selection using summary data from genome-wide association studies. Bioinformatics. 2016;32(10):1493–1501.

8. Millard LA, Munafò MR, Tilling K, Wootton RE, Davey Smith G. MR-pheWAS with stratification and interaction: searching for the causal effects of smoking heaviness identified an effect on facial aging. PLoS Genetics. 2019;15(10):e1008353.
